# SARS-CoV-2 receptor-binding domain deep mutational AlphaFold2 structures

**DOI:** 10.1038/s41597-023-02035-z

**Published:** 2023-03-14

**Authors:** Oz Kilim, Anikó Mentes, Balázs Pál, István Csabai, Ákos Gellért

**Affiliations:** 1 Department of Physics of Complex Systems, Eötvös Loránd University, Budapest, Hungary; 2 Wigner Research Centre for Physics, 1121 Budapest, Hungary; 3 Veterinary Medical Research Institute, Eötvös Loránd Research Network, 1581 Budapest, P.O. box 18, Hungary

**Keywords:** Machine learning, Molecular modelling, Nanocrystallography, Data publication and archiving

## Abstract

Leveraging recent advances in computational modeling of proteins with AlphaFold2 (AF2) we provide a complete curated data set of all single mutations from each of the 7 main SARS-CoV-2 lineages spike protein receptor binding domain (RBD) resulting in 3819*X*7 = 26733 PDB structures. We visualize the generated structures and show that AF2 *pLDDT* values are correlated with state-of-the-art disorder approximations, implying some internal protein dynamics are also captured by the model. Joint increasing mutational coverage of both structural and phenotype data coupled with advances in machine learning can be leveraged to accelerate virology research, specifically future variant prediction. We hope this data release can offer assistance into further understanding of the local and global mutational landscape of SARS-CoV-2 as well as provide insight into the biological understanding that 3D structure acts as a bridge between protein genotype and phenotype.

## Background & Summary

The receptor binding domain (RBD) of the SARS-CoV-2 spike protein, in its active conformation, is the domain that binds directly the to ACE2 receptor which itself is a protein on the surface of many cell types that acts as a “cellular doorway” for the SARS-CoV-2 virus. Understanding the competitive binding between RBD, ACE2, mono, and polyclonal antibodies is core to assessing the potential evolutionary fitness of a given variant.

Deep mutational scanning (DMS) experiments^1^ make gathering biophysical (phenotype) values such as protein expression as well as RBD-ACE2 binding affinity for close mutants in parallel possible, further enabling mapping of the local evolutionary landscape of any antigen. However, the variant combinatorial space hugely expands with the number of mutations of a protein; $$1{9}^{x}\left(\begin{array}{c}n\\ x\end{array}\right)$$ where *x* is the mutational distance (*x* = 15 for Omicron BA1^2^) and *n* is the sequence length (201 for the RBD). So there are approximately 4.23*10^30^ “Omicron-distant” variants from the original Wuhan sequence. DMS experiments produce data orders of magnitude too small to fully cover this number of possible mutants. Omicron for example, with its 15 mutations from the original Wuhan variant is a prime example of a more distant variant that has been shown to escape many antibodies evoked by early vaccines. It would have been hugely valuable to know this ahead of time which motivates the development of *in-silico* machine learning techniques to predict biophysical values with regard to the stability of proteins, expression extent, and most importantly protein-protein interaction Gibbs-free energies: Δ*G*.

**Fig. 1:**
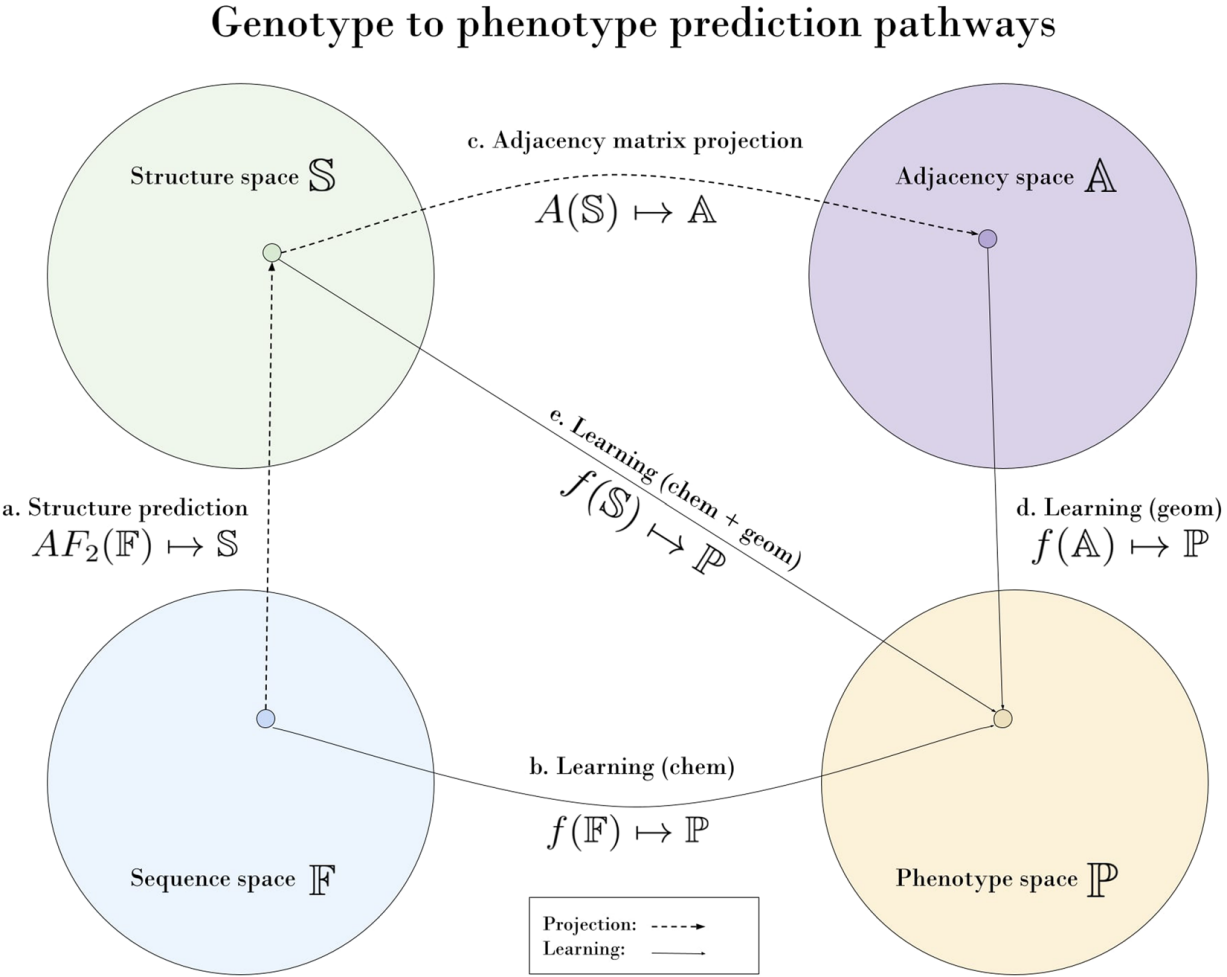
Sketch of protein representations and their projections. Sequence space 𝔽, structure space 𝕊, adjacency matrix space 𝔸, phenotype space ℙ are the spaces of all possible proteins for a given representation. 𝕊 contains 𝔽 and 𝔸, formally, 𝔽, 𝔸 ⊂ 𝕊. Each protein has a FASTA one-hot-encoded representation *F* ∈ 𝔽, a PDB file *S* ∈ 𝕊, an adjacency projection of the PDB file *A* ∈ 𝔸 and some measured phenotypic properties (function) *P* ∈ ℙ. We compare the projections 𝔽 and 𝔸 with respect to how a model *f* learns from these representations to make predictions about ℙ. (**a**) Predict structure with AlphaFold2.(**b**) Learning to predict protein-protein binding affinities from FASTA sequences. In the limit of huge amounts of genomic and phenotype data, this may even build such a rich internal representation of protein interaction dynamics that explicit structure modeling (the top path of the loop) is not required^41^. (**c**) Creation of adjacency matrices from PDB structures. Representations in A carry no chemical information so can be used to analyze if the AF2 projection to S actually captured geometric signal that can be leveraged for phenotype prediction tasks, this representation has the added advantage of being rotation agnostic. (**d**) Learning to predict protein-protein binding affinities with adjacency matrices. (**e**) 𝕊 representations in PDB contain both chemical and geometrical information. An end goal could be to use this representation to build predictive models to predict ℙ in a similar fashion to previously proposed methods^42–44^. However, this pathway is only worth using if we validate that (**d**) is possible to some extent.

Classical predictive models with hand-crafted features exist for the genotype to phenotype prediction problem^3,4^. However, the recent advanced in Supervised machine learning (ML) has led to models that often outperform classical models in presence of enough training data. This is due to their ability to learn task-specific features. Supervised machine learning as a tool fits the problem description where we have some distribution we want to learn and we require generalization to new unseen data which in our case would be unseen combinatorially distant variants. In Fig. 1 we outline how ML-based genotype to phenotype predictive models may be designed based on different representations of proteins. Proteins manifest as 3D folded forms natively so the 3D structure of proteins strongly dictates their biophysical properties. In order to leverage this prior knowledge in an ML framework we must gather 3D structures of proteins that match measured biophysical data to create pairs (*X, Y*) for model training where *X* is the 3D protein or protein-protein complex structure and *Y* is the corresponding biophysical measurement. Such a predictive model could be summarised as *f* where *Y* = *f*(*X*) and the loss ℓ(*Y*_*measured*_, *Y*_*predicted*_) is minimized with some minimization procedure. Such a model would allow vaccine developers to investigate how new antibodies/mixtures could be potent against future variants as well as allow the scientific and political community to get ahead of the virus evolution and be in a proactive position, not a reactive one that we are in at the time of writing. Bloom *et al*. have provided a preliminary tool^5^ based on a simple linear model to predict antibody escape (an important assessment of viral threat), however, this may deteriorate for more distant mutants. Predictive profiling of SARS-CoV-2 variants by deep mutational learning^6^ aims to produce a similar result but is limited by its 3D model-free nature. 3D model representations are only worth leveraging for predictive models if they contain additional signal that truly is physically relevant on top of the amino acid sequence loaded with chemical information. This motivated our study.

The ability of AlphaFold2^7^ (AF2) to produce accurate predictions for single mutants is under debate. Variable methodologies and datasets^8–12^ are used to make claims that predicted structures correctly resemble their measured counterpart. This is potentially difficult to assess as the effect of a mutation may be small compared to the inherent conformational dynamics and disorder of a given protein. In ref. ^9^ the authors chose specific illustrative examples of selected proteins for which experimental and structural data for both wild-type (WT) and structure-disrupting mutations are available. The authors compare the root mean squared distance (RMSD.) between structure disrupting mutations to the WT both for measured structures and AF2 predicted mutants. AF2 was unable to predict when a point mutation causes defective protein folding as both RMSD and *pLDDT* values were not concordant. This study however is impossible to perform for large sets of variants where there are no PDB ground truth reference structures so its conclusions are limited in ability to generalize to other proteins. In ref. ^10^ the authors find no correlation between Δ*pLDDT* values and biophysical measurement values of GFP fluorescence in 976 mutations of 90 proteins from the Thermo-Mut database^13^. The authors argue that some correlation between phenotypic values that relate to ΔΔ*G* and Δ*pLDDT* should be observed to indicate that generated AF2 single mutants possess a physically meaningful structure.

On the other side of the debate, ref. ^11^ leverage large-scale DMS data of 33 proteins with 117,135 mutations by investigating the correlations between the DMS predicted protein function values with both AF2 and experimentally derived structures. These protein phenotype quantities were calculated with structure-based protein function predictors; FoldX^14^, Rosetta^15^ and DynaMut2^16^. Strong correlations were seen for both experimentally derived structures and AF2-generated structures. It is, however, not possible to know if structure-based protein function predictors actually leverage 3D signal or simply use distilled sequence information to make their predictions. Additionally, in ref. ^12^ the authors show local structural change (as quantified with the local distance difference test (*LDDT*)^8^ metric) is correlated in experimental (PDB) and AF2-predicted pairs. Linking structure to phenotype as a data validation approach they find significant correlations between local structural changes in AF2-predicted structures and three categories of phenotype; fluorescence, folding, and catalysis across multiple experimental data sets.

This debate is still open and general validation of all structures generated by AF2 is out of the scope of this paper. Generalization of the model to new regions of the structural space may be very difficult to globally validate. At the time of writing, the utility of predicted 3D models needs to be evaluated on a case-by-case basis. In this spirit, we aim to validate SARS-CoV-2 RBD deep mutational AF2 structures which exist on a small manifold of this space. Despite this debate, we believe our validation methodology is well supported by the literature. The data set we release should help speed up research in the field of structural virology. This is because accessibility to our curated dataset alleviates resource and time burden for data generation and organization. This data can be used for downstream tasks for example predicting biophysical qualities or antibody escape^17^. Because we validate a diverse set of generated mutants, this validation should generalize to other RBD variants, not in our data release that researchers may want to generate themselves with AF2. In this work, we present a curated and validated dataset “SARS-CoV-2 RBD deep mutational AlphaFold2 structures”. Namely, 26733 aligned PDB structures to accelerate SARS-CoV-2-related research.

## Methods

### Generation of PDB library

We used the Ampere01 machine at the Wigner Research Centre for Physics (256 CPUs - AMD EPYC 7742 64-Core Processor, 8 nVIDIA A100 80GB GPU cards). A 5 TB SSD was used for the AlphaFold2 database access time acceleration. The input FASTA sequences were generated by first downloading the original spike protein (NCBI RefSeq: YP_009724390.1, UniProt ID: P0DTC2^18^) sequence and choosing the RBD section amino acids 331–531. From this sequence, we created the 6 other main mutants using the given positions and amino acid changes in Stanford University Coronavirus Antiviral Resistance Database^2^. This data is openly available. This was cross-validated against the main variant sequences^19^ and the GitHub repository from the Bloom lab where the complementary phenotypic data is hosted and experimental details of data generation is described^1^. The WT + 6 variant sequences were then iteratively mutated to create every possible single mutant. The RBD consists of 201 amino acid residues so the number of single mutants per variant is 201 × 19 = 3819. We will refer to this as a mutant cluster. This represents the positional range 331–531 of the full spike protein. The 7 mutants clusters produced were wuhan, alpha, beta, delta, eta, omicronBA1, and omicronBA2 amounting to 7 × 3819 = 26733 RBD protein FASTA sequence^20^ and structure files. These outputs contain all metadata files that were generated during AlphaFold2 runs. The running time for one RBD model was between 15 and 20 minutes. 5 structures were produced from each variant FASTA file and the model with the highest overall confidence was chosen for our library resulting in 7.2 GB for the entire library. We optimized the distribution of AlphaFold2 running 40 parallel jobs simultaneously. The selected structures were then aligned to the RBD part of the 6M0J RBD-ACE2 protein structure (PDB ID: 6M0J_2|Chain B[auth E] (15:208)^21,22^, UniProt ID: P0DTC2 (334:526)^18^) from the protein data bank with the Schrödinger Maestro built-in “structalign”^23^ post-processing method to both 6M0J and each cluster to its aligned parent variant as described in the Data Records section. All atoms are used for this alignment, this software uses an optimization method to minimize the RMSD between the two protein structures.

## Data Records

The datasets^24–30^ can be downloaded at: https://figshare.com/projects/SARS-CoV-2_RBD_single_mutant_AlphaFold2_structures/150089 or automatically downloaded and reprocessed with our script.n of our results. Each single mutant cluster will then be found within its variant folder under a folder named “structures”. For each variant, there are 19 randomly selected single mutants given as examples, and do not need to be unzipped. These files can be inspected immediately with any PDB reading software for example^31^. These files are automatically removed when using the./data_prepare.sh script to avoid duplicates in the final unzipped folders that the user can access. The filename of each PDB is provided in the structure:

*structures/{VARIANT}/rot-{VARIANT}_RBD_331_531_{reference allele}{residue position in Spike protein}{alternative allele}.pdb*. For example: *“structures/alpha/rot-Alpha_RBD_331_531_A344C.pdb”*.

We provide all generated sequence^20^. These are the inputs to the AF2 model. FASTA files are in the structure: *FASTA/{VARIANT}/{VARIANT}_RBD_331:531_{reference allele}{residue position in Spike protein}{alternative allele}.fasta*. For example: *“FASTA/alpha/Alpha_RBD_331:531_A344C.fasta”*.

We provide a re-aligned version^32^ of the same data set where firstly each main variant is aligned to the 6M0J^21,22^ structure and then all single mutant structures within each cluster were aligned to their respective parent variant structure. This may offer more flexibility for users of the data in exploring the potential complexes with the RBDs. Re-use or re-distribution of the data is compliant with the Attribution 4.0 International (CC BY 4.0) license.

## Technical Validation

### Visualizing generated data

**Fig. 2:**
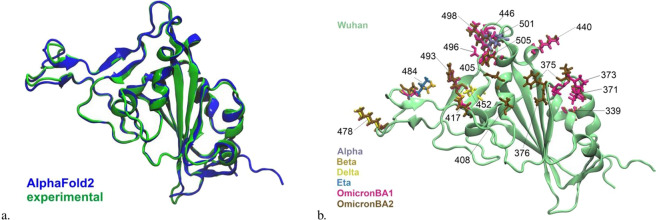
(**a**) AF2 aligned Wuhan WT RBD superimposed onto the experimentally determined 6M0J^21,22^ (RBD-ACE2 complex) clearly shows excellent agreement with respect to local and global structure. The RMSD value is 0.67 Å, which is due to the slight deviation between the structures in “loop” areas such as positions 371 and 478. (**b**) Variant defining mutations on SARS Cov-19 spike protein RBD. Wuhan RBD is in the cartoon illustration while the variant-defining mutations are illustrated with the licorice drawing method. The residue positions and the color codes are indicated.

**Fig. 3:**
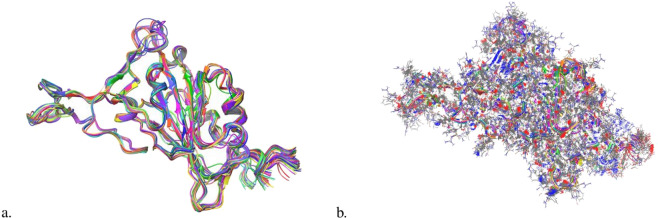
(**a**) Visualization of the entire cluster of single mutants backbones from Wuhan WT. Variation is observable however global overlap is clear. (**b**) Visualization of the entire cluster of single mutants from Wuhan WT with side chains visible. Diversity in positions is more prominent than looking at the backbone variation.

**Fig. 4:**
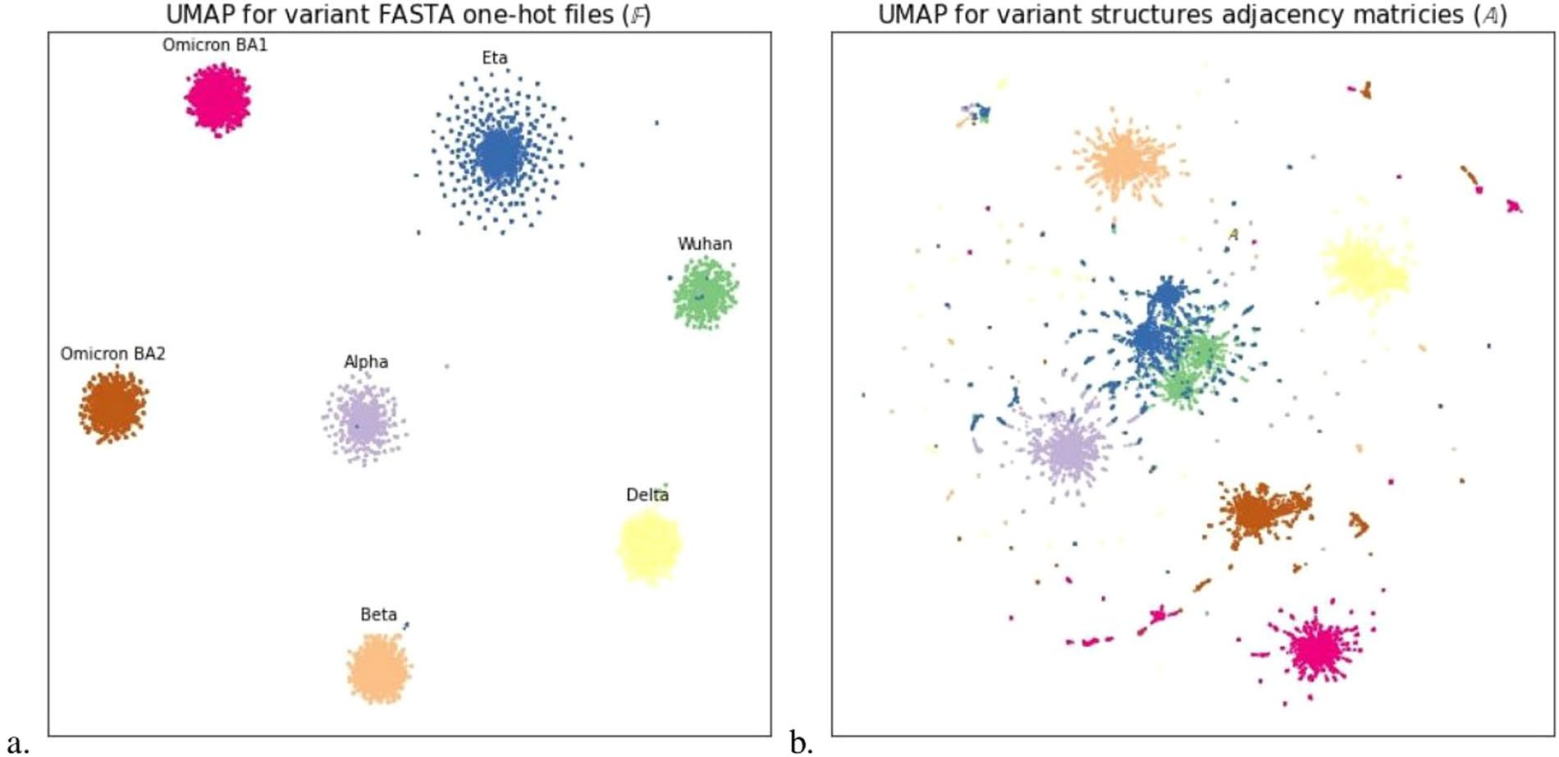
(**a**) UMAP^45^ embedding of the one-hot encoding representation from FASTA files. Distinct clusters are seen for each variant with homogeneous spacing in 𝔽 (**b**) UMAP embedding for all adjacency matrices. We can see similar clusters are conserved in the 3D structural data, however, there is more overlap between clusters in 𝔸 (the space of adjacency matrices, see Fig. 1c), indicating structural similarity between some variants. These embeddings also offer insight into the higher dimensionality and complexity of the generated structural information. We observe small sub-clusters (in 𝔸) of some lineages where distortions have taken place at the core of the structure causing more structural distortion, this may also cause drastic phenotypic change.

In Figs. 2, 3 we present aligned AF2-generated RBD variants. These images provide a first insight into the alignment and quality of the AF2 predictions in the region of the structure homologous we are investigating. In Fig. 4 we provide a visualization of how the representations of the entire library are related and are embedded in their respective spaces: 𝔽 and 𝔸 (see Fig. 1). In 𝔽 sequence space we see discrete clusters. Importantly in 𝔸 structural space we still see these clusters meaning signature information of all the structures belonging to one variant linage are somewhat conserved after the AF2 projection to structure space 𝕊 and then to adjacency matrix space 𝔸. In summary, variants’ chemical and geometric information are clustered independently.

### Validation of protein disorder

**Fig. 5:**
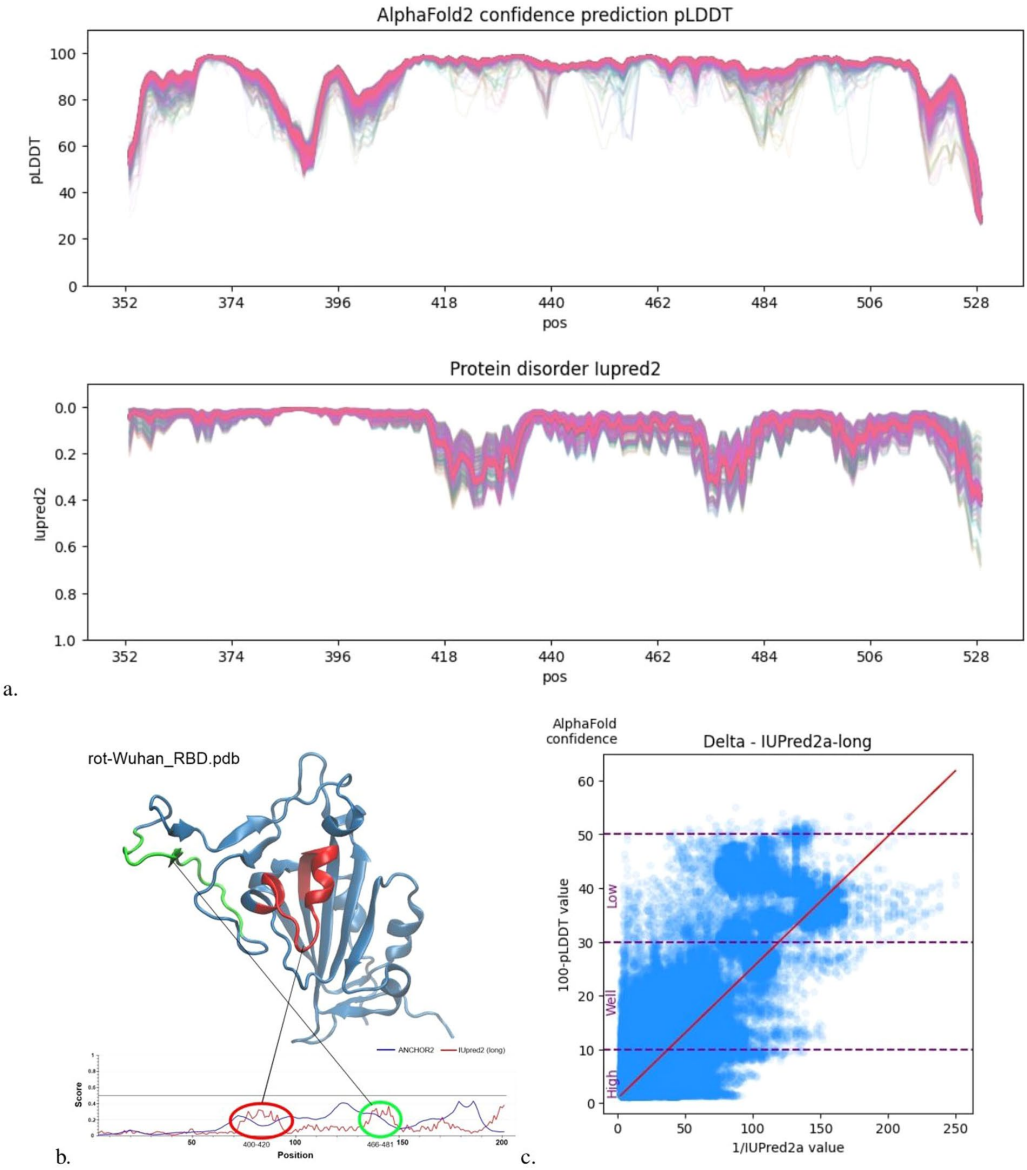
(**a**) Amino acid-wise protein disorder analysis for the Wuhan single mutants. In the upper diagram, discrete “valleys” are observed that are common to all single mutants. This elucidated the physical consistency of AF2 predictions in a single mutational cluster. The thick pink line is observed due to the extensive overlap of many of the variants *pLDDT* and *IU pred*2 values. (**b**) Structural intuition into high IUPred2 values. More disordered positions are at loops shown in red and green. (**c**) Transformed axes with Delta cluster as an example. A strong correlation is observed between the AF uncertainty measurement *pLDDT* and the *IU pred*2 disorder prediction. See Table 1.

As an investigation into the physical reliability of the AF2 predictions we explored the *pLDDT* scores generated for the variants^24–30^. Recent literature suggests *pLDDT* scores predicted by the model are correlated with disorder metrics^12,33,34^. Using the state-of-the-art disorder calculator IUPred2^35^ we find that 100-*pLDDT*∝1/*IU pred*2 for each cluster of single mutants (see Table 1 and Fig. 5). AF2 structures encoding relevant disorder information is critical specifically for SARS-CoV-2 variants as there is evidence that mutations preferentially emerge at intrinsically disordered protein sites^36^.

**Table 1:** |*R*^2^| values between 1/*IU pred* and 100-*pLDDT*.

Method^35^	Alpha	Beta	Delta	Eta	OmicronBA1	OmicronBA2
Short	0.44	0.42	0.49	0.47	0.58	0.73
Long	0.44	0.42	0.5	0.49	0.69	0.75

Relationship means *pLDDT* AF2 outputs capture internal protein disorder. Start and end 20 amino acids are ignored for this analysis as their disorder is only observed when protein is generated in-silico outside the context of the entire spike protein where it is bound. This is the case for all generated structures.

### Validation of 3D alignment

All structures we release are aligned to the RBD part of the 6M0J RBD-ACE2 protein structure^21,22^ from the protein data bank with the Schrödinger Maestro built-in “structalign” post-processing method. All RMDS values for these alignments can be downloaded from our figshare page^37^ All atoms have been involved in the structure alignments. The goal of this is to allow for standardization of the data set. This is beneficial for downstream modeling for example docking with protein partners and antibodies. We validate the quality of our alignment with the rotation agnostic dense adjacency matrix (contact map) representations of each variant A (See Fig. 1 for context).

1 $${\rm{A}}=\left[\begin{array}{ccccc}{d}_{11} & \ldots  & {d}_{1j} & \ldots  & {d}_{1n}\\ \vdots  & \ddots  & \vdots  & \ddots  & \vdots \\ {d}_{i1} & \ldots  & {d}_{ij} & \ldots  & {d}_{in}\\ \vdots  & \ddots  & \vdots  & \ddots  & \vdots \\ {d}_{n1} & \ldots  & {d}_{nj} & \ldots  & {d}_{nn}\end{array}\right]$$

Where ${d}_{ij}=\sqrt{{({x}_{i}-{x}_{j})}^{2}+{({y}_{i}-{y}_{j})}^{2}+{({z}_{i}-{z}_{j})}^{2}}$, using the residue center position: $x,y,z={\sum }_{n=1}^{{n}_{atoms}}\left(X,Y,Z\right)$ and *n* = 201 for the spike protein RBD. Structures that are more distorted have a larger adjacency matrix distance from their parent adjacency matrix. This distance corresponds only to the amount of distortion of the mutant and as adjacency matrices are independent of alignment. This is because each adjacency matrix value corresponds to an inter-residue-residue distance which does not change on structure rotation. Mutants that are more structurally distorted (have a larger adjacency matrix distance to their parent) can be expected to be more “difficult” to align. We found that these same proteins have a larger structalign RMSD. This validates the structalign alignment as, if the alignment was not good this observed correlation would be washed out. For example, highly distorted structures may not necessarily have the largest structalign RMSD and vice versa. There is a very strong correlation (R = 0.962) between variant RMSD from WT and Structure-based RMSD post-alignment. This should give users of the released data confidence in PDB alignments for further 3D modeling tasks.

## Usage Notes

### Complementary phenotype measurements for structures

The structures can be compared with phenotypic values measured by the Bloom lab. Full experimental details are also published^38^. All mutants can be matched by filename to the CSV file entry. These measurements include ACE2 binding and protein expression relative to their given parent WT. Some mutants have nan values in places where no reliable experimental results were possible to publish and may need to be removed or ignored for downstream tasks.

### PQR and APBS generation

We have prepared a script PQR-APBS.ipynb which can be used to transform all the structures to PQR^39^ and then APBS^40^ electrostatics form. The combined charge and structure features may offer complementary signal for downstream learning tasks.

## Data Availability

All code and instructions needed to reproduce all representations and results in the paper can be found at: https://github.com/csabaiBio/RBD-AlphaFold2-structures-and-phenotypic-information The GitHub repository contains scripts for: • https://github.com/csabaiBio/RBD-AlphaFold2-structures-and-phenotypic-information/blob/main/data_usage_scripts/aa_changes_vs_RMSD.ipynb Exploring simple amino acid variation statistics. • https://github.com/csabaiBio/RBD-AlphaFold2-structures-and-phenotypic-information/blob/main/interface_exploration/interface_importance.ipynb Exploring the importance of the interface with respect to ACE2 binding values. • https://github.com/csabaiBio/RBD-AlphaFold2-structures-and-phenotypic-information/blob/main/projections/UMAP_all_vars_structs.ipynb Visualizing embeddings. • https://github.com/csabaiBio/RBD-AlphaFold2-structures-and-phenotypic-information/blob/main/disorder_analysis/iupred_notebook-Analyses_mod.ipynb Investigating how pLDDT (b-factor) output of the AF2 files correlate with state-of-the-art disorder estimations.
